# Development of a Tailored Sol-Gel Immobilized Biocatalyst for Sustainable Synthesis of the Food Aroma Ester *n*-Amyl Caproate in Continuous Solventless System

**DOI:** 10.3390/foods11162485

**Published:** 2022-08-17

**Authors:** Corina Vasilescu, Cristina Paul, Simona Marc, Iosif Hulka, Francisc Péter

**Affiliations:** 1 Biocatalysis Group, Department of Applied Chemistry and Engineering of Organic and Natural Compounds, Faculty of Industrial Chemistry and Environmental Engineering, Politehnica University Timisoara, Carol Telbisz 6, 300001 Timisoara, Romania; 2 Faculty of Veterinary Medicine, Banat’s University of Agricultural Sciences and Veterinary Medicine ‘The King Michael I of Romania’, Calea Aradului 119, 300645 Timisoara, Romania; 3 Research Institute for Renewable Energy, Politehnica University Timisoara, Gavril Musicescu 138, 300501 Timisoara, Romania

**Keywords:** *Candida antarctica* B lipase (CalB), sol-gel entrapment, response surface methodology (RSM), green biocatalysis, food aroma ester production, continuous flow synthesis

## Abstract

This study reports the synthesis of a hybrid sol-gel material, based on organically modified silanes (ORMOSILs) with epoxy functional groups, and its application in the stabilization of lipase type B from *Candida antarctica* (CalB) through sol-gel entrapment. The key immobilization parameters in the sol-gel entrapment of lipase using epoxysilanes were optimized by the design of numerous experiments, demonstrating that glycidoxypropyl-trimethoxysilane can allow the formation of a matrix with excellent properties in view of the biocatalytic esterifications catalyzed by this lipase, at an enzyme loading of 25 g/mol of silane. The characterization of the immobilized biocatalyst and the correlation of its catalytic efficiency with the morphological and physicochemical properties of the sol-gel matrix was accomplished through scanning electron microscopy (SEM), fluorescence microscopy (FM), as well as thermogravimetric and differential thermal analysis (TGA/DTA). The operational and thermal stability of lipase were increased as a result of immobilization, with the entrapped lipase retaining 99% activity after 10 successive reaction cycles in the batch solventless synthesis of *n*-amyl caproate. A possible correlation of optimal productivity and yield was attempted for this immobilized lipase via the continuous flow synthesis of *n*-amyl caproate in a solventless system. The robustness and excellent biocatalytic efficiency of the optimized biocatalyst provide a promising solution for the synthesis of food-grade flavor esters, even at larger scales.

## 1. Introduction

Esters containing non-chiral alcohols and short-chain fatty acids are important additives used in the food and cosmetic industries and are particularly popular due to their fruity flavor [1]. Current processes for the production of esters consist of the esterification of a carboxylic acid with an alcohol in the presence of non-selective inorganic catalysts at high temperatures or via extraction from natural sources. Esters extracted from plant materials are often either too scarce or too expensive for commercial use, while those produced by chemical synthesis are not considered natural products [2]. Due to the growing concerns regarding climate change and environmental issues, industries are increasingly focusing on developing greener, safer, and more sustainable alternatives for the actual industrial-scale manufacturing processes [3]. Enzyme-catalyzed processes occur under mild reaction conditions, ambient temperature, atmospheric pressure, and physiological pH, therefore, being more environmentally friendly and cost-effective [4]. Compared to chemical catalysts, enzymes are readily biodegradable and non-toxic, reducing the hazards of processing [5]. These properties allow the manufacturing of quality products with fewer raw materials and lower chemicals, water, and energy consumption, and with less waste generation [6].

Interfacial active enzymes, such as lipases, can effectively catalyze not only hydrolytic but also synthetic reactions. Esterification reactions catalyzed by lipases are of great interest [7]. In particular, *n*-amyl caproate is an aroma chemical with a fresh floral flavor and a fruity apple taste with melon notes, marketed by several companies as a GRAS (Generally recognized as safe, as indicated by FEMA, the Flavor and Extract Manufacturers Association) flavoring compound accepted as an additive in different food products [8]. Consequently, numerous attempts have been made to develop an efficient lipase system for the synthesis of food-grade esters, showing that the influence of reaction parameters varies strongly with both substrate types. Therefore, each ester synthesis can be considered as a specific task [2]. To date, most lipase-catalyzed synthesis reactions are carried out in non-polar solvents, one of the main reasons for this being the homogeneity of the reaction mixture, even at lower operating temperatures. However, the actual requirements for sustainability and compatibility with food regulations have oriented the developments toward the utilization of green solvents or solventless systems. Biobased solvents from cereal or sugar sources are mainly obtained by the fermentation and valorization of lignocellulose residues, such as the furfural derivative, 2-methyl-tetrahydrofuran (2-MeTHF), or cyclopentyl methyl ether (CPME). Of these, 2-MeTHF is probably the most promising green solvent, obtained from carbohydrates and produced on an industrial scale from lignocellulosic biomass, particularly from agricultural wastes such as corn stover or sugarcane bagasse [9]. In recent years, several applications of 2-MeTHF, as well as CPME in biocatalysis have been reported [10]. Although organic solvents provide important advantages as reaction media for enzymatic reactions, in the case of the synthesis of food-grade flavor esters, the complete elimination of solvents is highly desirable [11]. Higher selectivity and volumetric productivity, along with improved substrate and product concentrations, are other advantages of using solventless reaction systems. Moreover, the production costs generally decrease by eliminating complex purification steps and provide a “clean” and “green” synthetic pathway by reducing the process hazards, accompanied by solvent exposure, toxicity, and flammability, shifting the manufacturing process toward an environmentally friendly route [12].

Immobilized lipases are excellent biocatalysts for the enzymatic synthesis of those short- and medium-chain fatty esters used as food flavor compounds; however, their catalytic activity depends greatly on the selected immobilization method [1]. Although numerous studies refer to lipase immobilization, there are still no straightforward protocols regarding the optimal method for each type of enzyme. Thus, it is necessary to customize the immobilization procedure for the selected enzyme and the envisioned applications. The choice of the appropriate immobilization procedure has to be evaluated considering the characteristics of the biomolecules, the supports, and the intended application. The uniqueness and versatility of the sol-gel immobilization process are provided by the mild, easy, and tunable synthesis conditions [13]. The sol-gel polymer network is compact enough to retain the enzyme molecules while allowing the substrate and products to pass through. The enzyme is not bound to the polymer matrix, so the inactivation of the enzyme is reduced, offering very high applicability [14]. The three-dimensional network of a sol-gel matrix creates a protective microenvironment for enzyme stability, enhanced by the forces involved in the support-enzyme interaction, such as electrostatic and hydrophobic interactions, which greatly influence enzyme performance in the case of a biocatalyst [7]. The great variety of organic silane precursors available allows many ways to improve the properties of the silica network. The most common and widely used precursors are tetraalkyl orthosilicates (tetraalcoxysilanes) Si(OR)_4_ (R being methyl or ethyl) in a mixture with trialkoxysilanes R′Si(OR)_3_ or dialkoxysilanes R′R′′Si(OR)_2_, substituted with alkyl or aryl functional groups (R′ and R′′), which allow the tuning of the xerogel matrix properties [15,16]. The properties of sol-gel particles are governed by the type and content of the particular organically modified silanes used [13]. Silanes with epoxy groups have multiple uses as coupling agents, even in the case of chemically modified 3D-printed scaffolds for enzyme immobilization [17], but their possible utilization as silane precursors for the sol-gel entrapment of lipases was reported only in our previous work [1]. The utilization of a precursor silane with an active epoxy group could lead to the supplementary stabilization of the enzyme by covalent binding without a severe multipoint attachment, due to the relatively low epoxy group content in the sol-gel matrix and their rather low reactivity under immobilization conditions. The covalent immobilization of enzymes on epoxy-activated supports allows a multipoint attachment via a reaction with the nucleophile groups of specific amino acid (lysine, histidine, tyrosine) residues that are located on the surface of the enzyme molecule, combined with physical adsorption [18]. Such supports are commercially available, but it is obviously difficult to control the interaction between the enzyme and the support, and inactivation can occur due to conformational changes induced by a too-strong multipoint binding. Therefore, the development and optimization of a sol-gel immobilization procedure, involving the entrapment of the enzyme and possible additional covalent bonding, is a challenging task.

The design of experiments (DOE) is an important approach when optimizing chemical processes [19], one that is used to effectively explore the relationships between the inputs and outputs of a process and to better understand them [20]. At the same time, it is increasingly used for the improvement of biocatalytic processes [21,22,23], but the optimization of immobilization parameters using this method is seldom reported [16]. Response surface methodology (RSM) is an optimization technique that is also used in biocatalysis and determines the optimum process conditions by testing several variables simultaneously [2].

The aim of this study was to investigate the influence of the innovative functionalization of the sol-gel matrix using silane precursors with epoxy groups, which could also allow improved stabilization by additional covalent bonding, on the catalytic performance of immobilized lipase from *Candida antarctica* B (CalB). The main novelty of this research is the utilization of an immobilized biocatalyst obtained via sol-gel entrapment, optimized through experimental design in the synthesis of the natural food aroma ester *n*-amyl caproate, in both batch and continuous systems. The best biocatalyst provided excellent thermal and operational stability in batch conditions, enabling a clean synthesis process and allowing further development toward a continuous-flow system. Moreover, the optimization of the major reaction parameters, substrate concentration, flow, and temperature in solventless conditions was accomplished, using, for the first time, a desirability approach to correlate the ester yield and productivity. These results demonstrate that a commercially marketed natural aroma ester can be synthesized in a continuous solventless process, using a novel biocatalyst obtained through an epoxy-functionalized sol-gel system and an immobilization process optimized via experimental design.

## 2. Materials and Methods

### 2.1. Materials

Lipase from *Candida antarctica* type B, produced by the fermentation of genetically modified microorganisms, was a generous gift from Genofocus (Daejeon, Republic of Korea). The commercial preparation CalB-IM^TM^ (Genofocus, Daejeon, Republic of Korea) contains lipase from *Candida antarctica* B, adsorbed onto a microporous ion-exchange resin. The silane precursors used for the sol-gel entrapment of the native lipase were tetramethoxysilane (TMOS) from Acros Organics (Geel, Belgium), while the silanes with epoxy functional groups, (3-glycidoxypropyl)trimethoxysilane (GPTMS) 99+% (product code SIG5840.1), (3-glycidoxypropyl)bis(trimethylsiloxy)methylsilane (GP(TMS)_2_MS) 97% (product code SIG5820.0) and 1,3-bis(glycidoxypropyl)tetramethyldisiloxane ((GP)_2_TMDSO) 97% (product code SIB1115.0) were purchased from Gelest (Morrisville, PA, USA). The other materials used were tris-(hydroxymethyl)-aminoethane, 2-propanol, 1-octyl-3-methyl-imidazolium tetrafluoroborate (OmimBF_4_), *n*-amyl alcohol, caproic acid, *n*-hexane, Coomassie Brilliant Blue G-250, bovine serum albumin (BSA), sodium fluoride, potassium fluoride, *n*-dodecane > 99%, fluorescein isothiocyanate (FITC), acetone, and 2-methyltetrahydrofuran (2-MeTHF), which were purchased from Merck.

### 2.2. Optimized Lipase Immobilization by Sol-Gel Entrapment

#### 2.2.1. Screening of Silanes with Epoxy Groups and Catalysts for Improved Sol-Gels

The immobilization of lipase B from *Candida antarctica* was studied by means of entrapment in hybrid sol-gel matrices that consisted of binary silane systems of tetramethoxysilane (TMOS) with an epoxysilane. In the process of optimizing the immobilization by sol-gel entrapment, several parameters were considered. First, the nature of the silane system and the type of the basic catalyst for the hydrolysis reaction of the silane, which greatly influence the properties of the enzymatic preparations, were investigated. For each of these two parameters, three distinct settings were chosen. In search of an optimal sol-gel network for enhanced enzyme activity, a combination of the three epoxysilanes with glycidoxypropyl groups, namely, GPTMS, GP(TMS)_2_MS, and (GP)_2_TMDSO with TMOS (at a 1:1 molar ratio), were tested. For each silane combination, three catalysts (sodium fluoride, potassium fluoride, and ammonia) were investigated.

The influence of the studied parameters (silane system and catalyst type) on the final properties of the immobilized preparations were evaluated in terms of immobilization yield and catalytic efficiency in the esterification reaction of *n*-amyl alcohol and caproic acid in a solventless medium.

#### 2.2.2. Statistical Optimization of Sol-Gel Entrapment of *Candida antarctica* B Lipase

*Candida antarctica* lipase type B was immobilized through sol-gel entrapment, using a binary TMOS:GPTMS system at different molar ratios between the two silanes. The influence of immobilization parameters, such as silane molar ratio (respectively, the organic matter content of the sol-gel network), and the enzyme loading (expressed as enzyme amount per total silane concentration) on the ester yield was investigated. The experimental setup was built using the Design-Expert software (Stat-Ease, Inc., Minneapolis, MN, USA), and process optimization was carried out by response surface methodology (RSM) employing a central composite design (CCD). The levels of the immobilization parameters investigated are given in Table S1 in the Supplementary Materials. All immobilization reactions were carried out in duplicate; the results given represent the means. Table 1 provides an overview of the experimental setup with 2 factors at 3 levels, consisting of the 11 immobilization runs (8 non-center and 3 center points) and the corresponding experimentally determined ester yields. The fit of the model to the experimental data was first evaluated, followed by an analysis of variance (ANOVA) to determine the statistical significance attributed to the model terms (immobilization parameters). Subsequently, the response equation for the quadratic model was given and the reaction parameters were optimized in order to maximize ester yield. Finally, data predictions were made using the fitted model, and a validation run was conducted under the optimized reaction conditions.

#### 2.2.3. Immobilization Procedure of *Candida antarctica* B Lipase and Immobilization Yield

The general procedure for the immobilization by sol-gel entrapment of enzymes, which was used as the starting point for the experimental design, was previously reported by the authors of [24]. Briefly, a certain amount of the *Candida antarctica* lipase B, depending on the enzyme loading used in the experiment, was suspended in Tris/HCl buffer (0.1 M, pH 8.0) and stirred magnetically at 600 rpm (rotations per minute) at room temperature, until a homogenous enzyme suspension was obtained. The suspension was further centrifuged at 15 °C and the supernatant was used for immobilization. The enzyme suspension, immobilization additives—ionic liquid, OmimBF_4_ and 2-propanol, and 1M solution of the hydrolysis catalyst (NaF/KF/NH_3_)—were added to a 4- mL glass vial and the mixture was kept under continuous stirring at room temperature. After 30 min, the appropriate silane precursors were added to the mixture in specific ratios (amounting to a total of 6 mmoles). The mixture was kept under stirring until gelation commenced and the obtained gel was kept at room temperature until complete polymerization was achieved. The wet gel was washed consecutively with 2-propanol, distilled water (for removal of unreacted compounds and unbound enzyme), 2-propanol, and hexane (for the removal of excess water), and vacuum-filtered through a glass Buchner funnel with a sintered filter disc of G3 porosity. Subsequently, the washed gel was dried for 24 h at room temperature and then in a vacuum oven at 25 °C for another 24 h (100 mbar final level of vacuum) for complete removal of the rinsing solvent. The resulting xerogel was crushed in a mortar and stored under refrigeration (4 °C).

The washing filtrate was tested for proteins using the Bradford method [25]. The efficiency of the immobilization procedure was evaluated in terms of protein immobilization yield, according to Equation (1):

(1) $$\mathrm{IY}=\frac{P_{i}}{P_{t}}\;\times \;100\;(\%),$$

where P_i_ is the immobilized protein quantity and P_t_ is the total protein quantity added in the immobilization protocol. P_i_ was determined by the subtraction of the unbound protein detected in the filtrate and washings from the initial total protein, P_t_. Although the Bradford protein assay results can be affected by several interferences, as was shown by Nicolas et al. [26], in our case, they were minimized by removing the insoluble components of the native solid CalB lipase and avoiding the utilization of phosphate buffers. In the conditions of sol-gel entrapment, the Bradford assay provides useful results, particularly when they are used for the comparative evaluation of different immobilization protocols.

A leaching test was also carried out to determine the possible loss of enzymes by diffusion in the solution. First, 20 mg of entrapped lipase was maintained for 1 h under stirring in 0.05 M phosphate buffer at a pH of 7.0; following centrifugation at 3000× *g*, a certain supernatant volume was used for the activity test of the possibly leached enzyme with *p*-nitrophenyl palmitate, as described by Guo et al. [27]. Enzymatic activity was not detected in the samples by this assay.

### 2.3. Catalytic Efficiency of the Immobilized CalB Lipase in Solventless Batch Esterification

The esterification reactions of *n*-amyl with caproic acid were carried out in a closed system in solventless media, as follows: in a 2-mL Eppendorf safe lock tube, substrates at a 1:1 molar ratio and with a native/immobilized enzyme, with a biocatalyst/substrate ratio of 9 and 50 (g/mol substrate) for the native and immobilized enzyme, respectively, were incubated in a Thermomixer (Eppendorf AG, Hamburg, Germany) at 36 °C and 1000 rpm for 16 h. The obtained ester amounts were assayed by gas chromatography, on a Varian 450 Chromatograph (Varian Inc., Utrecht, The Netherlands) equipped with a flame ionization detector (FID), using a 15 m × 0.25 mm VF-1ms non-polar capillary column with a 0.25-μm film thickness of dimethylpolysiloxane. The analysis conditions were as follows: oven temperature 80–160 °C, with a heating rate of 10 °C/min, injector temperature 300 °C, detector temperature 350 °C, and carrier gas flow (hydrogen) of 1.9 mL/min. The samples were dispersed in acetone, then a quantitative analysis was performed using *n*-dodecane as an internal standard. The ester yield and catalytic efficiency of the lipase were determined based on the GC data. The catalytic efficiency of the biocatalysts was expressed in terms of U/g biocatalyst, defined as the amount of ester (μmoles) synthesized per time unit (1 min) by 1 g of biocatalyst, under specific reaction conditions (36 °C, 1:1 substrate molar ratio, and 16 h reaction time). The term “efficiency” was utilized instead of “activity” because enzymatic activity is a property related to the rate of the enzyme-catalyzed reaction and would suppose a linear increase in the product amount during the whole reaction time (16 h in our case). The ester productivity expressed in g ester/g substrate (caproic acid) per time unit (1 h), obtained by converting 1 g of caproic acid using 1 g of biocatalyst under specific reaction conditions (36 °C, 1:1 substrate molar ratio, and 16 h reaction time), was also determined. All esterification experiments were run in duplicate, and the mean values were considered. Sample analysis was performed in triplicate.

### 2.4. Thermal Stability of the Biocatalysts

The sol-gel-entrapped biocatalyst CalB-SG and the native lipase CalB were incubated in *n*-amyl alcohol at different temperatures in the range of 40–80 °C. After 24 h, the biocatalysts were washed several times with acetone and centrifuged for substrate removal. Fresh substrates were added, and the biocatalyst’s activity was determined in the esterification reaction of *n*-amyl alcohol with caproic acid, as described in Section 2.3.

### 2.5. Influence of the Reaction Medium

For the green synthesis of the aroma ester *n*-amyl caproate, the reaction was tested in solventless conditions and in the presence of 2-methyltetrahydrofuran, the green alternative to the biomass-derived solvent, tetrahydrofuran. For comparison, we also studied the model esterification reaction of *n*-amyl alcohol with caproic acid in hexane media. The biocatalyst’s activity and ester yield were determined as described in Section 2.3.

### 2.6. Operational Stability of the Biocatalysts in the Batch Synthesis of n-Amyl Caproate

The sol-gel-entrapped biocatalyst CalB-SG, the native CalB lipase, and the commercial immobilized biocatalyst CalB-IM^TM^ were studied in repeated reuse cycles of the model reaction of *n*-amyl alcohol with caproic acid, as described in Section 2.3. After each esterification cycle, the solid biocatalyst was separated from the reaction mixture by centrifugation and washed several times with acetone. Fresh substrates were then added, and the reaction was run under the same conditions as previously described.

### 2.7. Characterization of the Biocatalysts

#### 2.7.1. Scanning Electron Microscopy (SEM)

Scanning electron microscopy was performed on a Quanta FEG 250 system (FEI, Hillsboro, OR, USA) using a secondary electron detector (SED). Powder samples were collected using a spatula and were fixed on SEM stubs with carbon tape. Studies were carried out in low vacuum mode at 5 kV and at 1.5 spot sizes, to avoid the charging of powder particles.

#### 2.7.2. Fluorescence Microscopy (FM)

Fluorescence microscopy was performed with an inverted microscope, the Leica DMI4000B (Leica, Munich, Germany), to investigate the distribution of the enzyme within the sol-gel nanostructures. For this purpose, the lipase from *Candida antarctica* B was dyed with fluorescein isothiocyanate (FITC), as described in the Pierce^TM^ FITC labeling kit. The removal of unbound FITC from the obtained solution was carried out by centrifugation in a centrifuge tube with an Amicon Ultra-4 filter (10 KDa cut-off) and repeated washings with distilled water until the absorbance of the collected fractions at the corresponding wavelength of 493 nm was approximately 0.1. The obtained solution (containing the FITC-labeled enzyme) was concentrated to 10 mg protein/mL by centrifugation in the Amicon Ultra-4 filter tube and was used for immobilization, as described in Section 2.2.3. For comparison purposes, a blank sol-gel matrix without the enzyme-FITC complex was also prepared.

#### 2.7.3. Thermal Analysis (TGA/DTA)

Thermogravimetric measurements (TGA/DTA) were recorded using a TG 209 F1 Libra thermogravimetric analyzer (Netzsch, Selb, Germany) operating at a resolution of 0.1 µg, in a nitrogen atmosphere. Thermogravimetric curves were recorded from 30 to 1000 °C, with a heating rate of 10 °C/min. The average sample mass was 4.0 ± 0.2 mg; the samples were tested in open alumina crucibles (average mass 190 ± 1.0 mg).

### 2.8. Continuous-Flavor Ester Synthesis in a Packed Bed Reactor in a Solventless System

The continuous synthesis of *n*-amyl caproate was achieved in a packed bed reactor (PBR) consisting of a stainless-steel column (dimensions: 150 × 4.6 mm) filled with approximately 1.5 g of sol-gel-entrapped *Candida antarctica* B lipase in consecutively stacked rows of biocatalyst and quartz sand (for improved hydrodynamics). Thus, a substrate solution was pumped through the bioreactor by means of an HPLC pump for controlled and constant flow rates. The stainless-steel column was placed in a thermostat, to maintain an adequate reaction temperature. The amount of ester synthesized per time unit was assayed periodically up to the 7 h mark by gas chromatography, to track ester formation and the stability of the system, as described in Section 2.3 for the batch reaction system. Sample analysis was performed in triplicate. The rate of production of the aroma ester, *n*-amyl caproate, was expressed in U/g and was defined as the amount of the ester (μmol) obtained per time unit (1 min) by 1 g of biocatalyst under specified reaction conditions. The productivity of the solventless system was expressed as the amount of g ester formed per hour and per g of the sol-gel biocatalyst.

## 3. Results and Discussion

### 3.1. Optimization of Lipase Immobilization by Sol-Gel Entrapment

#### 3.1.1. Screening of Silanes with Epoxy Groups and Catalysts for Improved Sol-Gels

The introduction of organic groups to the silica precursors leads to the so-called organically modified silica gels (ORMOSILs). While many studies have been published on the subject of organically modified silica gels, the relationship between the properties of the functionalized support and the enzyme activity is still not completely understood [13]. The catalytic efficiency of immobilized enzymes strongly depends on the local environment provided by the supporting material. Reetz et al. [28] pioneered the use of *n*-alkyltrimethoxysilanes as precursors in the sol-gel process, demonstrating their efficiency in the modification of the specific activity of immobilized lipases. The utilization of functionalized silane precursors for lipase immobilization is one of the most successful achievements of the sol-gel technique.

The organically modified alkoxide silane (3-glycidoxypropyl)trimethoxysilane (GPTMS) owns two distinct functional groups, methoxy, that can be hydrolyzed to silanol groups that form a silica network during the condensation process, and a pending epoxy group through which an organic network formation can be achieved [29]. The terminal epoxy group can easily undergo ring-opening reactions and can also be used as a coupling agent to covalently bind organic and inorganic compounds [30]. In our study, hybrid organic-inorganic sol-gel matrices incorporating epoxysilanes were investigated for the entrapment of lipases. Having highly reactive functional groups, we assumed that the epoxysilanes could also lead to the formation of covalent bonds with the protein molecules, allowing a more stable double immobilization of enzymes [1].

Another important factor in sol-gel formation is the nature of the catalyst. Specifically, a basic catalyst leads to larger pores in the sol-gel matrix, compared to acid catalysts [31]. For this reason, sol-gel immobilization was carried out with basic catalysts, such as sodium fluoride, potassium fluoride, and ammonia.

Furthermore, the presence of additives in the sol-gel matrix formation process can decrease the internal tension and contraction of the material during gel formation [31] and can significantly increase the catalytic activity of the immobilized enzymes [32]. Ionic liquids have been proven to increase the effectiveness of sol-gel entrapment [31]. In our previous study [24], the ionic liquid OmimBF_4_ was proven to enhance the activity and enantioselectivity of the immobilized enzyme.

**Figure 1:**
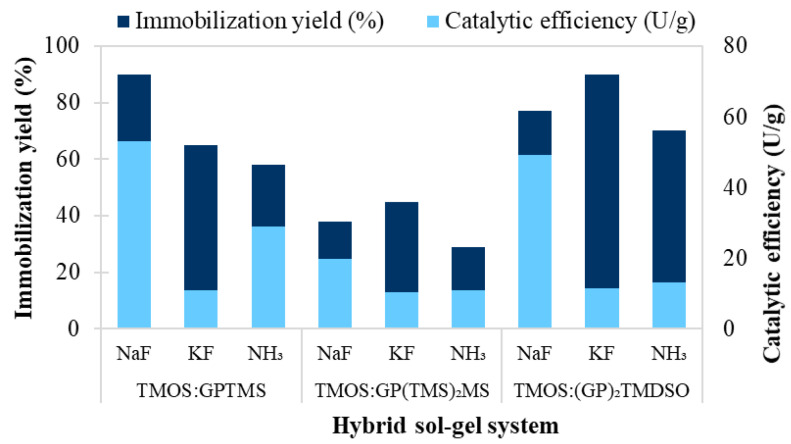
The influence of the silane system and catalyst type on the immobilization yield and catalytic efficiency of sol-gel-entrapped *Candida antarctica* B lipase.

Figure 1 reveals a strong correlation between the silane system of the sol-gel network and the catalytic efficiency of the entrapped biocatalyst. It can be noted that although the KF catalyst facilitates the entrapment of a larger amount of enzyme, especially in the case of networks containing larger silane molecules, such as GP(TMS)_2_MS and (GP)_2_TMDSO, a significant loss in the catalytic activity of the entrapped enzyme was observed. KF also seems to favor the formation of a tighter sol-gel network, leading to impeded mass transfer. Similarly, ammonia was also found to be a weak catalyst in terms of immobilization yield, as well as the catalytic activity of the entrapped enzyme. Interestingly, the TMOS:(GP)_2_TMDSO silane system, although capable of entrapping a large amount of enzyme due to the rather spacious structure of the epoxysilane, proved to be disadvantageous in terms of enzyme activity, except for the use of NaF as a catalyst. The results show that TMOS:GPTMS was the most advantageous silane network system when NaF was used as the catalyst.

According to these results, the best biocatalyst for the synthesis of the targeted aroma ester was obtained by entrapment in a sol-gel matrix, obtained with TMOS:GPTMS silane precursors at an equimolar ratio and with NaF as a catalyst.

#### 3.1.2. Statistical Optimization of Sol-Gel Entrapment of *Candida antarctica* B Lipase

A response surface methodology by means of a 3-level-2-factor central composite design was employed to optimize the immobilization parameters in the sol-gel entrapment of *Candida antarctica* B lipase, such as the silane molar ratio of the precursor silane system and enzyme loading. Table 1 provides an overview of the experimental setup, as well as the predicted and observed data of the yield of the esterification reaction catalyzed by the lipase, immobilized according to the results presented in Section 3.1.1.

Among the different immobilization reactions, the maximum ester yield (88%) was achieved in experiment no. 9 at a 2:1 silane ratio and 16.66 g/mol enzyme loading, while the minimum yield (only 28%) was recorded in experimental run no. 2, at the lowest enzyme loading value and the lowest organic content in the sol-gel network (3:1 silane ratio, 8.33 g enzyme/mol silanes). In all experiments, immobilization yields of between 78 and 90% were obtained.

*Model fit*

**Figure 2:**
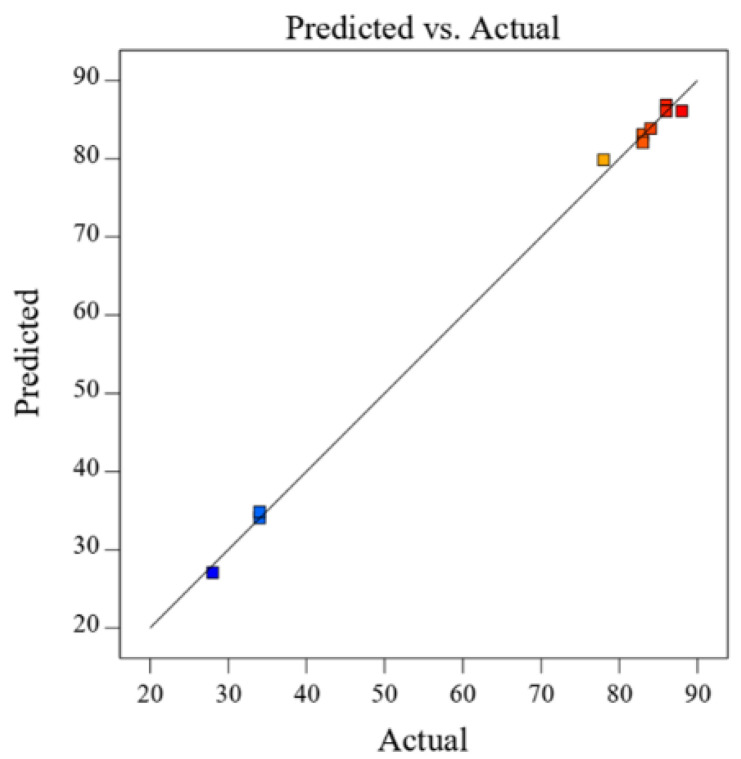
Correlation of the calculated (predicted) versus the experimental (actual) values for the ester yield in the synthesis of *n*-amyl caproate catalyzed by sol-gel-entrapped lipase from *Candida antarctica* B.

The fit of the quadratic model was examined using the R^2^ value and was determined to be 0.998, indicating that up to 99.8% of the variability in the response could be explained by the model. The plot of experimental values of ester yield (%), versus those calculated from the model equation (Figure 2), suggests a good fit between the data, with a correlation coefficient (predicted R^2^) of 0.989. This in reasonable agreement with the adjusted R² of 0.997. Overall, these results revealed that the predicted and experimental values are in good agreement, implying that the empirical model derived from RSM can be used to effectively define the relationship between the factors and the response of the system in the enzymatic synthesis of *n*-amyl caproate by sol-gel-entrapped *Candida antarctica* B lipase.

*Analysis of variance (ANOVA)*

Statistical analysis of the experimental data was performed using the Design-Expert software from Stat Ease, Inc. (Minneapolis, MN, USA) and allowed for the estimation of the main effects and interaction effects of the investigated immobilization parameters.

The statistical significance of the model terms was determined using an analysis of variance. The multiple regression coefficients were obtained by employing a least-squares technique to predict a second-order polynomial model Equation (2) for ester yield; the results of the statistical analysis are summarized in Table 2.

In comparison, the interaction term AB was found to be of less significance. Values greater than 0.1 indicate that the model terms are not significant. The model also showed a statistically insignificant lack-of-fit relative to the pure error, having an *F*-value of 1.87. Factor B (enzyme loading) has the greatest influence on the system studied, as also highlighted by the perturbation plot (Figure S1 in the Supplementary Materials). The final response equation obtained for the quadratic model was:

Ester yield = + 86.11 − 2.00 A + 26.00 B + 1.50 AB − 4.26 A^2^ − 25.26 B^2^ (2)

It seems that the most relevant variable for enzyme immobilization is enzyme loading, with estimated effects of 26.00, while the silane molar ratio has a slightly negative influence on the ester yield (−2.00). The results indicate the importance of working with high loads of the enzyme.

*Process optimization*

**Figure 3:**
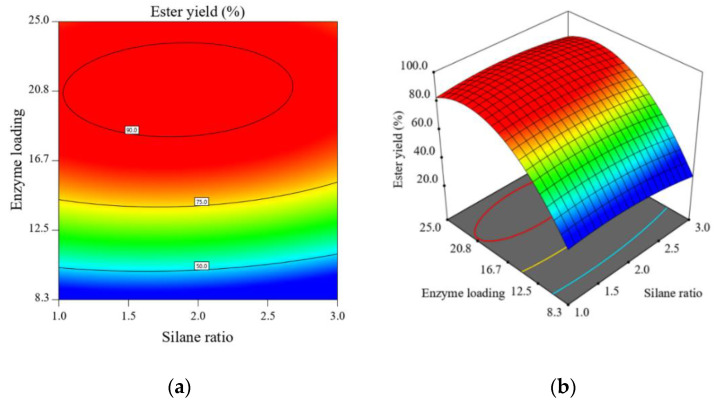
Contour (**a**) and 3D surface (**b**) plots of the system response (ester yield) in the enzymatic synthesis of *n*-amyl caproate, catalyzed by sol-gel-entrapped lipase from *Candida antarctica* B for different values of factors A (TMOS:GPTMS silane ratio) and B (enzyme loading).

Data were visualized using contour plots, as well as 3D surface plots. Graphical optimization plots are given in Figure 3. As discussed previously, the silane ratio seems to have little influence on the catalytic efficiency of the entrapped lipase; however, since the immobilization yields were lower at superior ratios, we concluded that lower ratios are preferable for the immobilization through sol-gel-entrapment of *Candida antarctica* B lipase. The optimization analysis revealed that maximum ester yields can be obtained for enzyme loadings above 20 g/mol silane precursors. Since the immobilization yields were generally below 90%, to account for the loss of enzyme in the immobilization procedure, we determined an enzyme loading of 25 g/mol of silane precursors as an optimum.

*Point prediction and model validation*

Given these points by statistical analysis and optimization, model validation was carried out at the suggested TMOS:GPTMS silane ratio of 1:1 and enzyme loading of 25 g/mol of silane precursors. The predicted value of ester yield was 83%, which is in good agreement with the experimentally observed value of 84%.

#### 3.1.3. Immobilization Procedure of *Candida antarctica* B Lipase and Immobilization Yield

The immobilization yields obtained for the entrapment of *Candida antarctica* B lipase in sol-gel matrices consisting of the binary silane system TMOS:GPTMS in different molar ratios and with different enzyme loadings are presented in Table 3. The immobilization procedure proved to be very effective, with high protein entrapment yields, the lowest yield obtained being 78% for the TMOS:GPTMS silane system at a 3:1 molar ratio and the lowest enzyme-loading.

The increase in organic content of the sol-gel network (TMOS:GPTMS silane ratio) led to significantly lower gelation rates and, accordingly, to an exponential rise in gel formation time, from a couple of seconds to 12 h (data not shown).

### 3.2. Catalytic Efficiency of the Immobilized CalB Lipase in Solventless Batch Esterification

The ester yield obtained in the enzymatic synthesis of *n*-amyl alcohol with caproic acid alongside the sol-gel-entrapped CalB lipase under optimized conditions (TMOS:GPTMS silane ratio of 1:1, and enzyme loading of 25 g/mol silane) was 84%, close to that obtained with the native enzyme (87%) under the same reaction conditions, demonstrating that the lipase was not inactivated during the immobilization process.

Pertaining to the catalytic efficiency of the CalB lipase (106 U/g), an expected decrease in the biocatalyst after immobilization was observed, which can be explained by the in-depth distribution of the enzyme throughout a much larger support matrix. However, it remained at satisfactory and economically viable levels (18 U/g), very close to those of the commercial enzyme preparation, CalB-IM^TM^ (20 U/g).

### 3.3. Thermal Stability of the Immobilized Biocatalyst

**Figure 4:**
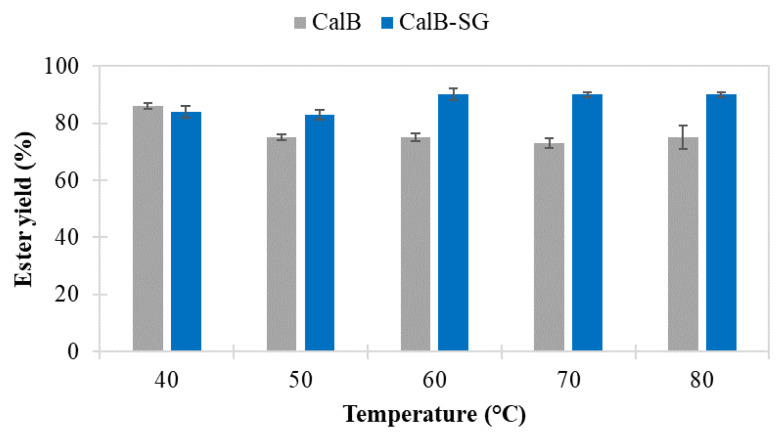
Influence of the preincubation of *Candida antarctica* lipase B (CalB—native lipase; CalB-SG—sol-gel-entrapped CalB lipase) at different temperatures on the ester synthesis yield of n-amyl caproate in a solventless medium.

The temperature stability of immobilized enzymes is one of the advantages of sol-gel entrapment because the conformational flexibility of the enzyme is reduced by encapsulation inside the xerogel matrix [15]. Thus, the temperature stability of the lipase from *Candida antarctica* B (CalB) was studied via preincubation in *n*-amyl alcohol (also used as the reaction substrate) at temperatures ranging from 40 to 80 °C. The influence of temperature on the ester yield is presented in Figure 4, compared to the native biocatalyst.

The activities of the sol-gel-immobilized lipase remained constant at all incubation temperature values, displaying excellent thermal stability in the studied temperature range of 40–80 °C. In contrast, the native lipase lost up to 15% of its activity as the incubation temperature increased to 80 °C. This same tendency was noticed concerning the catalytic efficiency values, which were almost constant in the range of 11–12 U/g for CalB-SG, while showing a slight decrease from 104 U/g at 40 °C to 84 U/g at 80 °C for the native CalB. The significant catalytic efficiency differences between the native and immobilized CalB lipases are due to the much lower enzyme amount that was effectively introduced in the reaction system, using an immobilized biocatalyst that contains only about 170 mg enzyme/g preparation, since a major part of the immobilized material is silica xerogel.

While both native and immobilized lipase have been used successfully over the tested temperature range, the immobilized enzyme proved more stable at elevated temperatures (80 °C) than the native lipase. The increase in incubation temperature had a beneficial effect on ester production when using the sol-gel-immobilized lipase, as can be seen from the slightly higher ester yields, probably due to improved mass transfer.

### 3.4. Influence of the Reaction Medium

**Figure 5:**
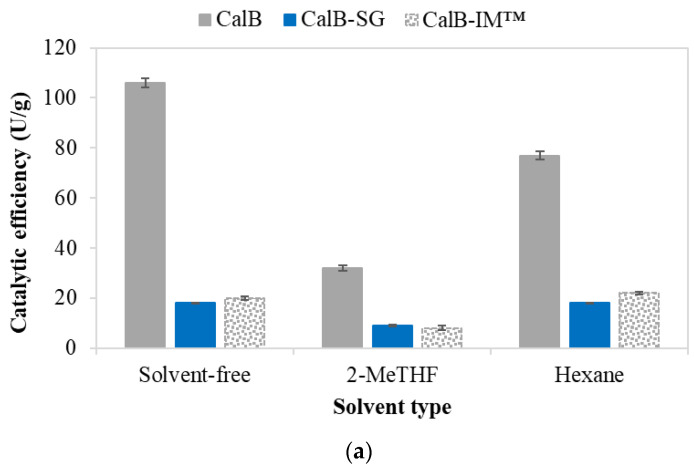

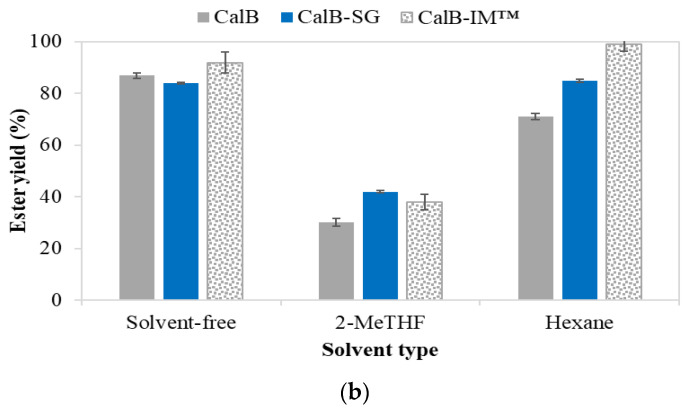
Influence of the reaction medium on the catalytic efficiency (**a**) and ester synthesis yield (**b**) of *n*-amyl caproate at 36 °C, catalyzed by *Candida antarctica* lipase B (CalB—native lipase; CalB-SG—sol-gel-entrapped CalB lipase).

Most of the esterification processes catalyzed by lipases are accomplished in non-polar organic solvents, considering that in this way the water can be displaced from the catalytic center zone, while the equilibrium is shifted toward esterification [33]. In the search for a greener synthesis method for the food aroma, ester *n*-amyl caproate, we carried out the reaction in both organic solvent and solventless systems. Along with *n*-hexane, the most commonly used non-polar solvent, the green biomass-derived solvent 2-methyltetrahydrofuran (2-MeTHF) was also tested. However, using this solvent for the esterification reaction significantly lowered catalytic efficiency (Figure 5a), and lower yields (Figure 5b) were obtained than in *n*-hexane or in the solventless medium. The commercially immobilized lipase CalB-IM™ exhibited similar behavior, losing more than 50% in terms of activity in 2-MeTHF, compared to *n*-hexane and the solventless system. The sol-gel-entrapped lipase demonstrated comparable catalytic efficiency with the commercial biocatalyst, proving that it could be used successfully for this process, also having the advantage of reusability in comparison with the native lipase. The reaction in the solventless system led to slightly higher yields, as in *n*-hexane, and allowed us to carry out subsequent experiments without any solvent.

### 3.5. Operational Stability of the Biocatalysts in the Batch Synthesis of n-Amyl Caproate

The most important feature of enzymes that can be improved by immobilization is their stability upon reuse in consecutive reaction cycles, a key requirement for industrial application. Resistance to mechanical and chemical degradation by solvents and/or substrates (alcohols and acids), as well as the compatibility of the sol-gel with the reaction medium (hydrophobic, in the case of *n*-hexane solvent, but more hydrophilic in solventless conditions, especially when using excess amounts of alcohol) were important features of the chosen immobilization technique. Moreover, since the designed sol-gel biocatalyst was further used for the continuous flow synthesis of *n*-amyl caproate in a packed-bed reactor, it was essential to maintain the integrity of the sol-gel matrix, not only under shear stress but also under the backpressure present in this type of reactor, to prevent enzyme leaching.

**Figure 6:**
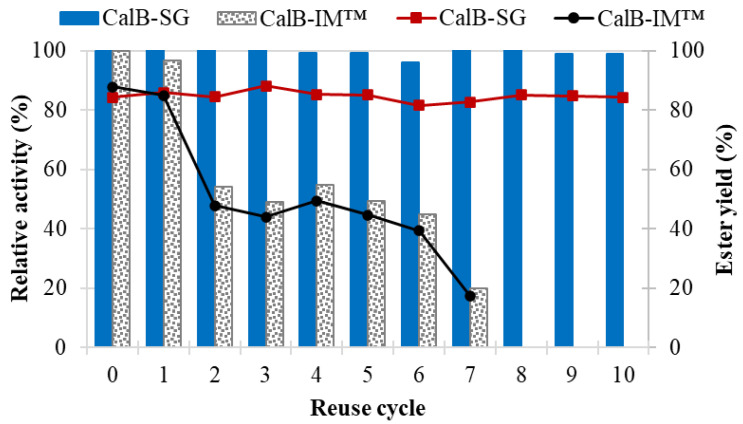
Operational stability of lipase from *Candida antarctica* B in successive batch reaction cycles of caproic acid esterification with *n*-amyl alcohol at 36 °C, in a solventless medium. The column diagram depicts the relative activities, while ester yields are shown in red (CalB-SG lipase, immobilized by sol-gel entrapment) and black (CalB-IM™ lipase, immobilized by adsorption onto ion exchange resin) line segments, respectively.

A reusability study was carried out to demonstrate that the immobilized biocatalyst is robust and maintains its stability over many reaction cycles. The operational stability of *Candida antarctica* B lipase, immobilized by an optimized sol-gel entrapment procedure using a binary system of silane precursors (TMOS:GPTMS in 1:1 molar ratio and an enzyme loading of 25 g enzyme/mol silane), was studied in repeated batch esterification cycles of the esterification reaction of *n*-amyl alcohol and caproic acid, in a solventless system. The results are shown in Figure 6, in comparison to the commercially available immobilized enzyme preparation, CalB-IM™, derived from the same lipase.

The lipase immobilized by the sol-gel technique proved its superiority, preserving more than 99% of its initial activity, even after 10 reaction cycles. Conversely, the relative activity of the commercially available CalB-IM^TM^ lipase decreased significantly under the selected process conditions, losing up to 80% of its initial value after 7 reuse cycles.

The ester yields in the solventless synthesis in batch mode showed the same tendency. In the case of CalB-SG, they were almost constant during the 10 reuse cycles, in the range of 82–88% ± 1.5%, while for the reference commercial biocatalyst, CalB-IM™, a decrease from 88% in the first cycle to 17% after 10 uses was noticed.

The catalytic efficiencies per g of biocatalyst that were achieved in the solventless synthesis of *n*-amyl caproate in the batch mode were 17.6–18.1 U/g biocatalyst, in the case of CalB-SG, and 3.9–19.2 U/g biocatalyst for the resin-adsorbed lipase CalB-IM™, respectively. The ester productivity relative to the amount of substrate was also determined, as described by Li et al. [34]. As there are two substrates in the esterification reaction, the results were expressed in relation to the natural caproic acid. As expected, the ester productivities in the repeated batch processes, were also in a close range for CalB-SG between 1.62 and 1.71 g ester/g acid during 10 batch reaction cycles. At the same time, the reference biocatalyst CalB-IM^TM^ exhibited a consistent decrease in productivity, dropping from 1.74 g ester/g acid to 0.35 g ester/g acid at the end of only 7 batch-reaction cycles.

The high reusability of the sol-gel-immobilized biocatalyst is mainly due to the inherent properties of silica gels. The xerogels obtained in the sol-gel immobilization process are poly-siloxane glasses with high mechanical and chemical stability, these being the main advantages of these systems compared to resins (as CalB-IM™) or other supports that are commonly used for the immobilization of enzymes [35].

### 3.6. Characterization of Optimized Sol-Gel-Entrapped Biocatalyst

#### 3.6.1. Scanning Electron Microscopy (SEM)

**Figure 7:**
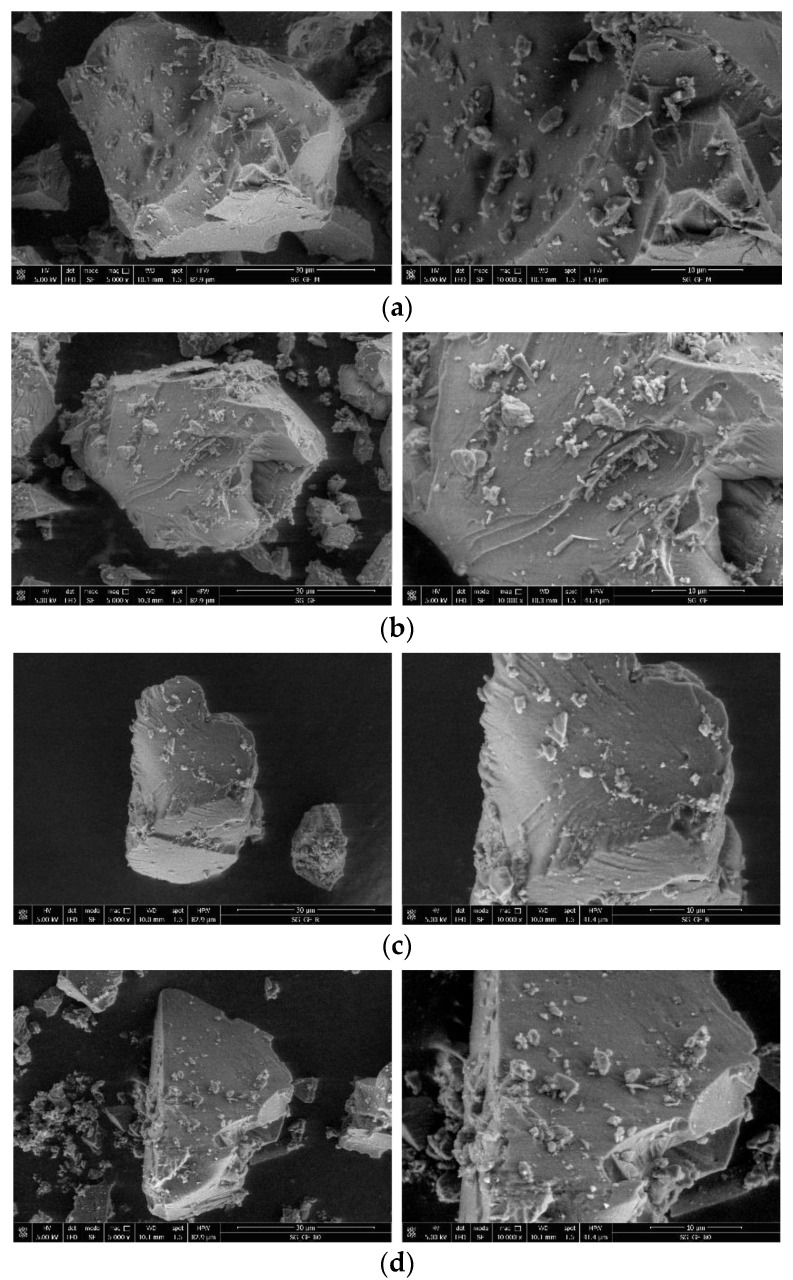
SEM micrographs at 5000× and 10,000× magnification of the: (**a**) blank sol-gel matrix; (**b**) sol-gel-entrapped *Candida antarctica* B lipase; (**c**) the recovered sol-gel biocatalyst after repeated use; (**d**) the recovered sol-gel biocatalyst after thermal stress.

The morphology and particle size distribution of the sol-gel-entrapped lipase were analyzed and measured using scanning electron microscopy. As described in the general procedure for immobilization by sol-gel entrapment (Section 2.2.3), the resulting xerogel was crushed in a mortar. The brittle bulk material was fragmented into smaller particles, which are generally in the range of 1–70 microns. The SEM micrographs are presented in Figure 7, at two different magnifications of 5000× and 10,000×, respectively.

SEM images of the CalB-SG sample presented in Figure 7b show particles that have an irregular shape and a wide size distribution in the range of 13–57 microns. The block shape morphology, with smooth surfaces and sharp edges, is characteristic of brittle fractures. In addition, tiny powder particles, generally smaller than 4 microns, can be seen on the surface of the larger particles. These small particles tend to form agglomerations, as can be seen from the micrographs. Similar findings were reported by Erasmus et al. [36].

The morphology of the blank sol-gel matrix (without enzyme) in Figure 7a is similar to the preparation containing the entrapped enzyme, with a particle size distribution in the range of 15–67 microns. For the CalB-SG samples recovered after reuse in more than 10 reaction cycles (Figure 7c), and after incubation in *n*-amyl alcohol at 80 °C for 24 h (Figure 7d), a similar morphology to the initial CalB-SG preparation was observed; it retains the block shape structure, which is in good correlation with the excellent activity demonstrated and remained constant after thermal stress and reuse. Only a small difference related to the edges of the particles could be detected. As can be observed in the SEM micrographs, the edges are less sharp and more rounded/smoother compared to the initial preparation, probably due to temperature stress or repeated usage. Furthermore, fine particles in the range of 15–20 microns are missing, which might be caused by the repeated washing of the powder during the recycling and reuse process.

#### 3.6.2. Fluorescence Microscopy (FM)

Fluorescence microscopy was used to observe the distribution of the enzyme within the sol-gel network. Since *Candida antarctica* B lipase does not possess inherent fluorescence, it was labeled with fluorescein isothiocyanate (FITC).

**Figure 8:**
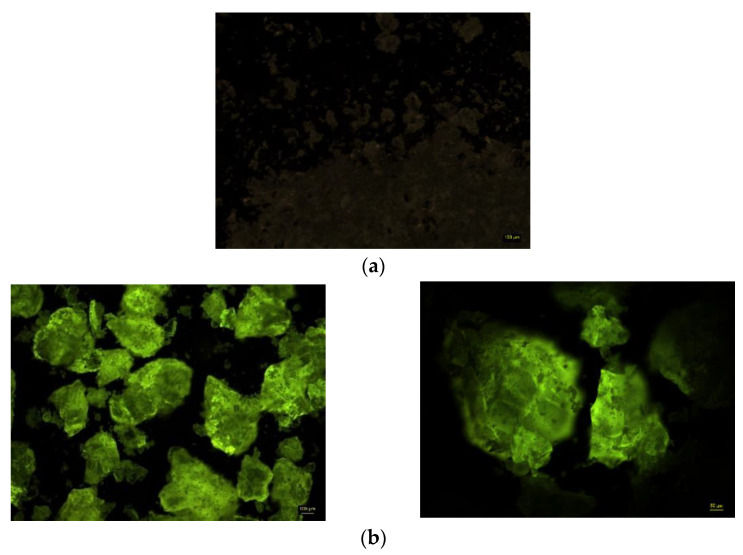
Fluorescent images of: (**a**) the blank sol-gel matrix (without enzyme) at 5× magnification; (**b**) the sol-gel matrix, containing FITC-labeled *Candida antarctica* B lipase at 5× and 10× magnification.

The fluorescent image of the FITC-enzyme complex that was immobilized in the TMOS:GPTMS matrix was recorded, as shown in Figure 8b, compared to the blank matrix in Figure 8a.

The image of the xerogel preparation containing the FITC-enzyme complex showed fluorescence (which is absent in the control xerogel), with the enzyme distributed on the surface and in the inner part of the sol-gel matrix, indicating that the enzyme is distributed uniformly throughout the immobilized preparation.

#### 3.6.3. Thermal Analysis (TGA/DTA)

**Figure 9:**
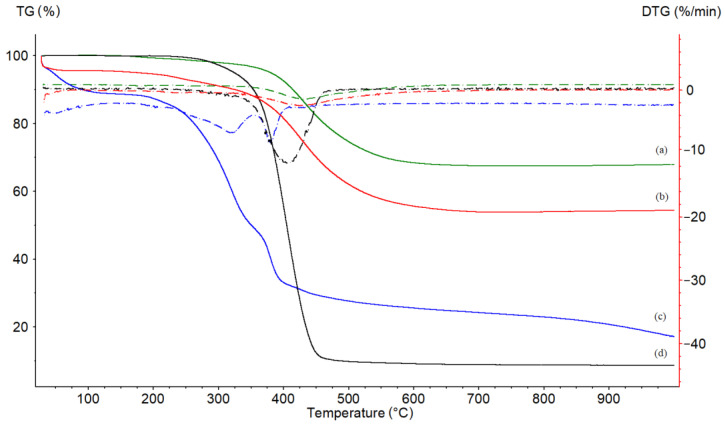
Thermograms showing the weight loss (TG, continuous line) and their derivative (DTG, dotted lines) for: blank sol-gel matrix (SG, green) (**a**); sol-gel immobilized *Candida antarctica* B lipase (CalB-SG, red) (**b**); unbound *Candida antarctica* B lipase (CalB, blue) (**c**), and commercial *Candida antarctica* B lipase preparation (CalB-IM^TM^, black) (**d**).

The weight-loss curve of the sol-gel-entrapped biocatalyst obtained by thermogravimetric analysis (TGA) is presented in Figure 9. The weight loss percentage of the three regions observed (30–280 °C, 280–530 °C, and 530–990 °C) is given in Table 4.

In the first region (30–280 °C), a weight loss of 1.7–5.2 is associated with the evaporation of water and some volatile compounds that are used in the sol-gel process. The sol-gel preparation containing the entrapped lipase showed a weight loss 3.6% higher than that of the matrix without enzyme, showing that there is a significant amount of water remaining in the preparation that is important for the preservation of the active catalytic conformation of the biocatalyst. This is correlated with the good biocatalytic efficiency obtained for our CalB-SG preparation. The difference of 6.1% in weight loss between the sol-gel preparation and the blank sol-gel matrix, SG, in the 280–530 °C interval can be assigned to the entrapped protein. The DTG curves of the CalB-SG and the blank SG matrix are quite similar, showing a peak at 450 °C compared to that of the native lipase at only 350 °C, indicating greater protection against increasing temperatures after immobilization, as shown in our previous studies [15,24].

The thermogram of the native lipase in Figure 9c was completely different in comparison with that of the sol-gel preparation (Figure 9b). The weight loss in the first region was higher (approximately 21.6%), showing the high water content of native lipase, even when the unbound lipase is a solid material. The thermal decomposition of the organic part of the native lipase is completed at 530 °C, showing a significant weight loss of 48.7%. The protein content measured by the Bradford method for the native lipase was 7%, the 41.7% difference representing organic compounds that were added to the lipase in order to stabilize it. The residual mass of 17.13% indicates the inorganic compounds added to the native lipase as stabilization additives. The commercial CalB-IM™ biocatalyst shows a significant weight loss of 89% to 530 °C (Figure 9d), meaning that the immobilization support is an organic ion exchange resin. Comparatively speaking, the sol-gel matrix is mostly inorganic.

### 3.7. Continuous Flavor Ester Synthesis in a Packed Bed Reactor in a Solventless System

The immobilization of *Candida antarctica* lipase type B by entrapment in epoxy-functionalized hybrid sol-gel led to enzyme loading of about 170 mg/g and protein loading of 13.3 mg/g, respectively.

**Figure 10:**
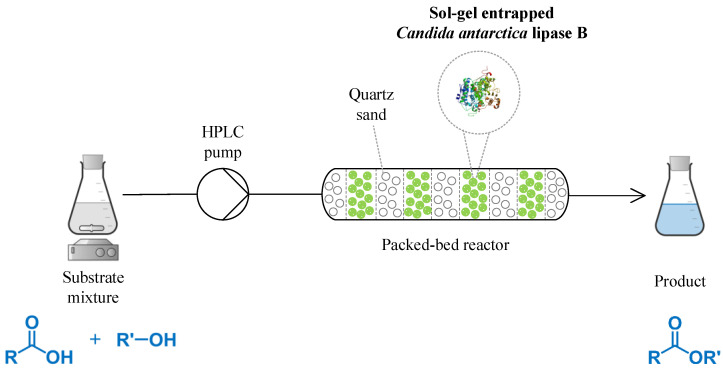
Continuous flow esterification of caproic acid and *n*-amyl alcohol, catalyzed by sol-gel-entrapped *Candida antarctica* B lipase (CalB-SG) in a solventless system.

The continuous flow esterification of caproic acid and *n*-amyl alcohol (Figure 10) was performed in a solventless system, using a packed-bed reactor with 1.5 g immobilized enzyme (20 mg of protein); the main parameters and their interactions in the enzymatic synthesis of *n*-amyl caproate in a continuous flow regime were evaluated. The investigated reaction parameters were substrate concentration, flow, and temperature, each at three distinct levels. The levels of the three esterification reaction parameters are given in Table 5.

**Figure 11:**
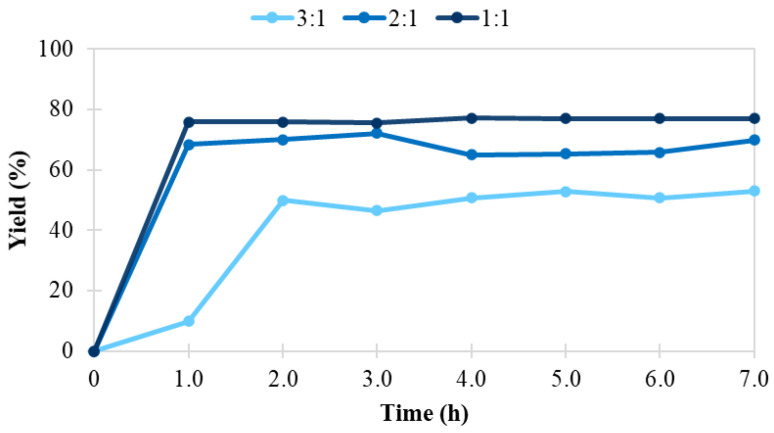
Ester yield (%) in the continuous enzymatic synthesis of *n*-amyl caproate in a solventless system, catalyzed by sol-gel-entrapped *Candida antarctica* B lipase at a flow rate of 0.2 mL/min, a temperature of 80 °C, and different substrate molar ratios.

**Figure 12:**
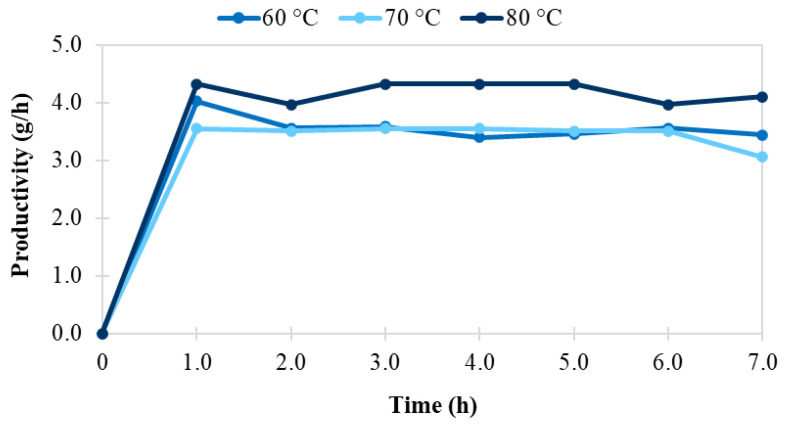
Productivity (g/h) in the continuous enzymatic synthesis of *n*-amyl caproate in solventless system catalyzed by sol-gel-entrapped *Candida antarctica* B lipase at a flow rate of 0.4 mL/min, a substrate molar ratio of 2:1 and different temperatures.

The system response was evaluated in terms of both ester yield (Figure 11 and Figure S2 in the Supplementary Materials) and productivity (Figure 12 and Figure S3 in the Supplementary Materials).

At the given enzyme/substrate ratio (0.03 g enzyme/g caproic acid, 3.38 g enzyme/mol caproic acid), a maximum ester yield of 77% was achieved at a substrate molar ratio of 1:1, flow of 0.2 mL/min, and temperature of 80 °C. Although the yield in the given conditions was not as high, we obtained excellent production rates (r_flow_ of 312 U/g), and productivities (3.5 g ester/h per gram of biocatalyst).

The maximum productivity of 4.15 g ester/h per gram of sol-gel biocatalyst, however, was obtained at elevated flow rates and slightly different reaction conditions: a substrate molar ratio of 2:1, flow of 0.4 mL/min, and temperature of 80 °C, pertaining to a maximum production rate of 370 U/g. Comparatively, Thomas et al. [37] obtained production rates of 250 U/g in the continuous flow kinetic resolution of secondary alcohols, and a conversion rate up to 57%.

It was also found that the ester yield did not vary over a period of 7 h for the synthesis of *n*-amyl caproate in the continuous flow packed-bed reactor, beyond the experimental error. Thus, we concluded that the catalyst efficiency did not diminish for 7 h of biocatalysis, even without the removal of water.

Concerning the production of aroma esters on a larger scale, the use of packed-bed reactors containing immobilized enzymes is more cost-effective than traditional batch-mode reactors. Research in the field of the miniaturization of packed-bed reactors has also revealed promising results, such as improved mass transport, leading to higher yields and shorter reaction times [38].

Table 6 shows examples of biocatalytic ester synthesis reactions that were carried out in different types of packed-bed reactors with immobilized enzymes, as reported by other groups, in comparison with our results.

Sheih et al. [39] investigated the synthesis of hexyl laurate in a packed-bed reactor (25 cm × 0.25 cm) for the esterification of lauric acid and 1-hexanol, catalyzed by immobilized *Rhizomucor miehei* lipase (Lipozyme IM-77, 1.5 g). They obtained high conversion rates (97%) using a 1:2 alcohol/acid substrate ratio in hexane solvent, at a flow rate of 4.5 mL/min and 45 °C. The maximum production rate was 437.6 μmol/min.

Subsequently, Sheih et al. [40] used *Rhizomucor miehei* lipase, immobilized by adsorption onto anionic resin (Lipozyme IM-77), in a packed-bed reactor for the synthesis of hexyl laurate in solventless media. Under optimal conditions (54 °C and a flow rate of 0.5 mL/min) at a lauric acid concentration of 0.3 M, they obtained an ester production rate of about 87 µmol/min and a maximal yield of 60%.

Woodcock et al. used Novozym 435 for the synthesis of alkyl esters in a miniaturized continuous-flow packed-bed reactor (30 cm × 1.65 mm). A substrate solution of 0.2 M in hexane (molar ratio of 1:1 acid/alcohol) was pumped through a packed bed of Novozyme 435 (approximately 100 mg) at 1 μL/min and 23 °C, yielding alkyl esters at conversions of > 99% over a two-hour period. They attained a productivity rate of 2.04 mg ester/hour with 100 mg of biocatalyst, at a flow rate of 1 μL/min [41].

Wang et al. [38] used a microfluidic chip packed bed reactor (1 cm × 500 μm × 75 mm) filled with 90 mg of Novozym 435 to synthesize caffeic acid phenylethyl esters (CAPE) by the transesterification of alkyl caffeates with phenylethanol, using ionic liquid BmimTf_2_N as the solvent. Under optimal conditions, they obtained up to 93% of ester yield at a flow rate of 2 μL/min and 2.5 h residence time. The maximum productivity achieved was 0.027 μmol/min at a flow rate of 20 μL/min, 60 °C, and 3 mg/mL substrate concentration (molar ratio of 1:40 ester/alcohol).

Although we did not surpass a substrate conversion of 77% with the given enzyme loading (0.02 g enzyme/g total substrate, at 1:1 molar ratio) and experimental setup, the obtained ester yields at this stage of the continuous flow experiments in the solventless enzymatic synthesis of the aroma ester *n*-amyl caproate offer very promising results and could potentially be further improved by the use of elongated packed-bed reactors and the longer residence time of the substrates.

We obtained very good ester production rates (up to 370 U/g) that were much higher than those usually achievable for ester synthesis reactions in solventless systems, comparable to those accomplished in solvent-entrained systems. For example, Shieh et al. obtained about the same amount of immobilized lipase and comparable flow rate, demonstrating much lower ester productivity in the solventless synthesis of hexyl laurate.

In addition, the productivities attained in our work are much higher than in the case of microchannel reactor systems, which benefit from improved mass transport and system performance.

## 4. Conclusions

*Candida antarctica* B lipase immobilization through entrapment with epoxysilanes was proven to be an improved sol-gel technique for rendering highly stable biocatalysts and appropriate for the clean production of food aroma esters, particularly *n*-amyl caproate, under mild reaction conditions (36 °C, solventless system). 

The stability of the biocatalyst was investigated under thermal stress and repeated usage, in comparison to the native enzyme and a commercial preparation of the same enzyme. Under the investigated conditions, the sol-gel biocatalyst exhibited excellent catalytic efficiency and robustness against external stressors. Furthermore, it proved high cost-effectiveness by maintaining 99% of its initial activity, even after 10 reuse cycles.The continuous flow synthesis of the aroma ester *n*-amyl caproate catalyzed by the entrapped CalB lipase was evaluated in terms of flow, temperature, and alcohol:acid molar ratio, leading to high productivity (4.15 g ester/h) at the optimal values of these parameters: a substrate molar ratio of 2:1, a flow of 0.4 mL/min, and a temperature of 80 °C, respectively.

## Figures and Tables

**Table 1:** Experimental setup with coded and experimental values of the immobilization parameters and system response.

Exp. No.	Coded Values		Experimental Values		Ester Yield, %	
	Factor A	Factor B	TMOS:GPTMOS Ratio	Enzyme Loading, g/mol	Observed	Predicted
1	−1	−1	1:1	8.33	34	34
2	+1	−1	3:1	8.33	28	27
3	−1	+1	1:1	25.00	83	83
4	+1	+1	3:1	25.00	83	82
5	−1	0	1:1	16.66	84	84
6	+1	0	3:1	16.66	78	80
7	0	−1	2:1	8.33	34	35
8	0	+1	2:1	25.00	86	87
9	0	0	2:1	16.66	88	86
10	0	0	2:1	16.66	86	86
11	0	0	2:1	16.66	86	86

**Table 2:** Analysis of variance for the quadratic model for ester yield (%).

Source	SS	df	MS	*F*-Value	*p*-Value
Model	6035.84	5	1207.17	594.2	<0.0001
A—Silane ratio	24	1	24	11.81	0.0185
B—Enzyme loading	4056	1	4056	1996.48	<0.0001
AB	9	1	9	4.43	0.0892
A^2^	46.04	1	46.04	22.66	0.0051
B^2^	1616.84	1	1616.84	795.85	<0.0001
Residual	10.16	5	2.03		
Lack-of-fit	7.49	3	2.5	1.87	0.3667
Pure Error	2.67	2	1.33		
Corr Total	6046	10			

**Table 3:** Immobilization yields (%) for the sol-gel entrapment of *Candida antarctica* B lipase.

Exp. No.	TMOS:GPTMOS Ratio	Enzyme Loading, g/mol Silane	Immobilization Yield, %
1	1:1	8.33	90
2	3:1	8.33	78
3	1:1	25.00	95
4	3:1	25.00	95
5	1:1	16.66	90
6	3:1	16.66	89
7	2:1	8.33	80
8	2:1	25.00	92
9	2:1	16.66	88
10	2:1	16.66	85
11	2:1	16.66	88

**Table 4:** Thermal behavior of the studied biocatalysts.

Biocatalyst	Weight Loss, %			Residual Mass, %
	30–280 °C	280–530 °C	530–990 °C	
CalB	21.62	48.75	9.35	17.13
Blank SG matrix	1.67	26.63	3.81	67.89
CalB-SG	5.26	32.80	4.70	54.43
CalB-IM™	1.40	89.02	0.91	8.62

**Table 5:** Experimental setup of the continuous enzymatic synthesis parameters of *n*-amyl caproate catalyzed by sol-gel-entrapped *Candida antarctica* B lipase, along with the system responses.

Experimental Factor	Level		
Flow, mL/min	0.2	0.3	0.4
Temperature, °C	60	70	80
Substrate (alcohol: acid) molar ratio	1:1	2:1	3:1

**Table 6:** Some examples of enzyme-catalyzed synthesis of esters in a continuous flow regime.

Ester	Biocatalyst	Immobilization	Reactor Type	Solvent Type	Ester Yield, %	r_flow_, U/g	Productivity, g/h	Ref.
*n*-amyl caproate	CalB	Sol-gel entrapment	PBR	-	77	370	4.15	this work
hexyl laurate	RM (Lipozyme IM-77)	Adsorption onto anionic resin	PBR	hexane	97	292	4.98	[39]
hexyl laurate	RM (Lipozyme IM-77)	Adsorption onto anionic resin	PBR	-	60	58	0.99	[40]
alkyl esters	CalB (Novozym 435)	Adsorption onto acrylic resin	PBR	hexane	>99	118	0.02	[41]
CAPE	CalB (Novozym 435)	Adsorption onto acrylic resin	MFC	IL	93	0.3	0.005	[38]

CalB: *Candida antarctica* lipase B; CRL: *Candida rugosa* lipase; RM: *Rhizomucor miehei* lipase; PBR: packed-bed reactor; MFC: microfluidic chip; CAPE: caffeic acid phenylethyl ester.

## Data Availability

The data presented in this study are available on request from the corresponding authors.

## Supplementary Materials

The following supporting information can be downloaded at: https://www.mdpi.com/article/10.3390/foods11162485/s1, Figure S1: Perturbation plot of experimental factors A (TMOS:GPTMS silane ratio) and B (enzyme loading) on the system response (ester yield, %) in the enzymatic synthesis of n-amyl hexanoate catalyzed by sol-gel-entrapped lipase from Candida antarctica B; Figure S2: Ester yield (%) in the continuous enzymatic synthesis of n-amyl hexanoate in solventless system catalyzed by sol-gel-entrapped Candida antarctica B lipase at: (a) a substrate ratio of 2:1 and a temperature of 60 °C; (b) a substrate ratio of 2:1 and a flow rate of 0.2 mL/min; Figure S3. Productivity in the continuous enzymatic synthesis of n-amyl hexanoate in a solventless system catalyzed by sol-gel-entrapped Candida antarctica B lipase at: (a) a substrate ratio of 2:1 and a temperature of 60 °C; (b) a temperature of 80 °C and a flow rate of 0.2 mL/min; Table S1: Immobilization parameters of sol-gel entrapment of Candida antarctica B lipase used in the CCD optimization setup.
