# Supplementary material for: Development of a Tailored Sol-Gel Immobilized Biocatalyst for Sustainable Synthesis of the Food Aroma Ester n-Amyl Caproate in Continuous Solventless System
Source: Foods. 2022 Aug 17;11(16):2485. doi: 10.3390/foods11162485 (PMC9407315; doi:10.3390/foods11162485)
Supplement: Supplementary file 1 [file foods-11-02485-s001.zip › foods-1850323-supplementary.pdf]

Development of a Tailored Sol-Gel Immobilized Biocatalyst for Sustainable Synthesis of the Food Aroma Ester *n*-Amyl Caproate in Continuous Solventless System

Corina Vasilescu [1], Cristina Paul [1,*], Simona Marc [1,2], Iosif Hulka [3], Francisc Péter [1,3]

[1] Biocatalysis Group, Department of Applied Chemistry and Engineering of Organic and Natural Compounds, Faculty of Industrial Chemistry and Environmental Engineering, Politehnica University Timisoara, Carol Telbisz 6, 300001 Timisoara, Romania

[2] Faculty of Veterinary Medicine, Banat's University of Agricultural Sciences and Veterinary Medicine 'The King Michael I of Romania', Calea Aradului 119, 300645 Timisoara, Romania

[3] Research Institute for Renewable Energy, Politehnica University Timisoara, Gavril Musicescu 138, 300501 Timisoara, Romania

* Correspondence: cristina.paul@upt.ro

Table S1. Immobilization parameters of sol-gel entrapment of *Candida antarctica* B lipase used in the CCD optimization setup

Parameter	Name	Units	Minimum	Maximum	Mean	Std. dev.
Factor A	Silane ratio		1:1	3:1	2:1	0.77
Factor B	Enzyme amount	g/mol	8.33	25.00	16.66	6.46
Response Y	Ester yield	%	28	88	70.00	24.59

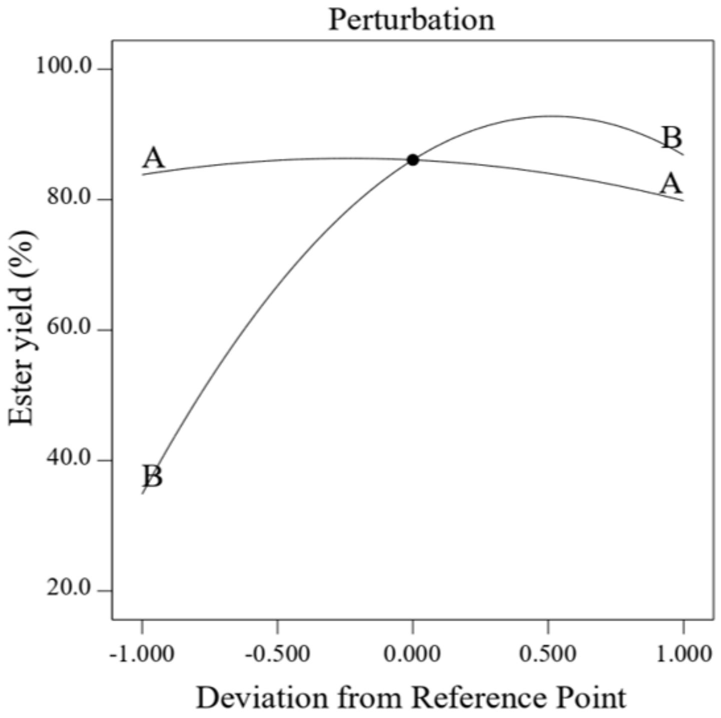

Figure S1. Perturbation plot of experimental factors A (TMOS:GPTMS silane ratio) and B (enzyme loading) on the system response (ester yield, %) in the enzymatic synthesis of *n*-amyl hexanoate catalyzed by sol-gel entrapped lipase from *Candida antarctica* B

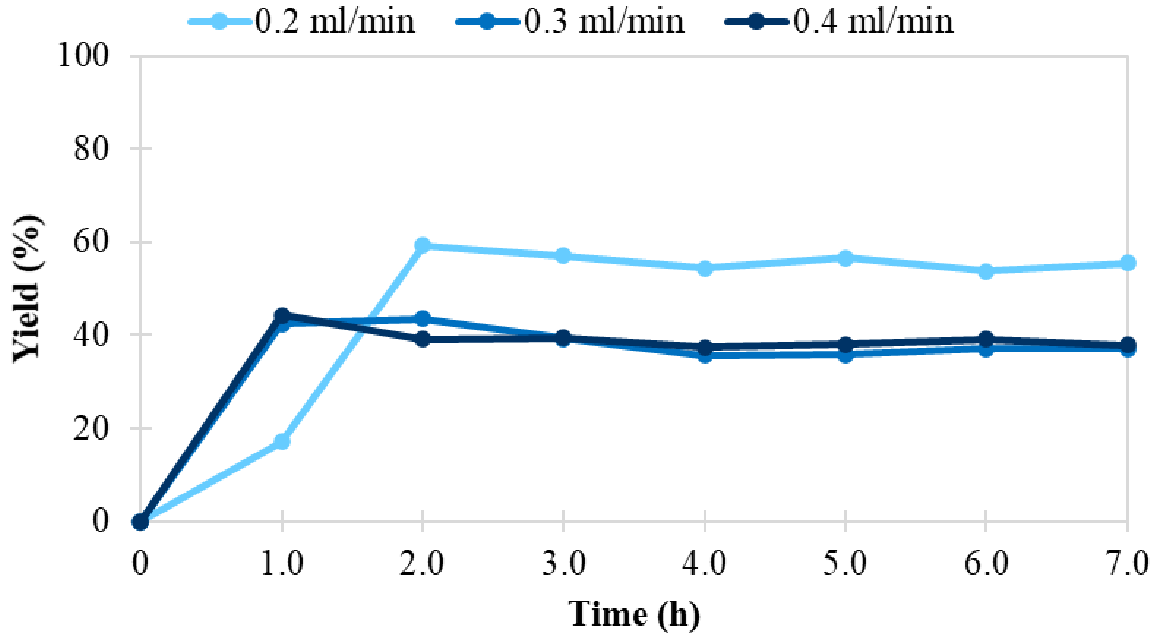

(**a**)

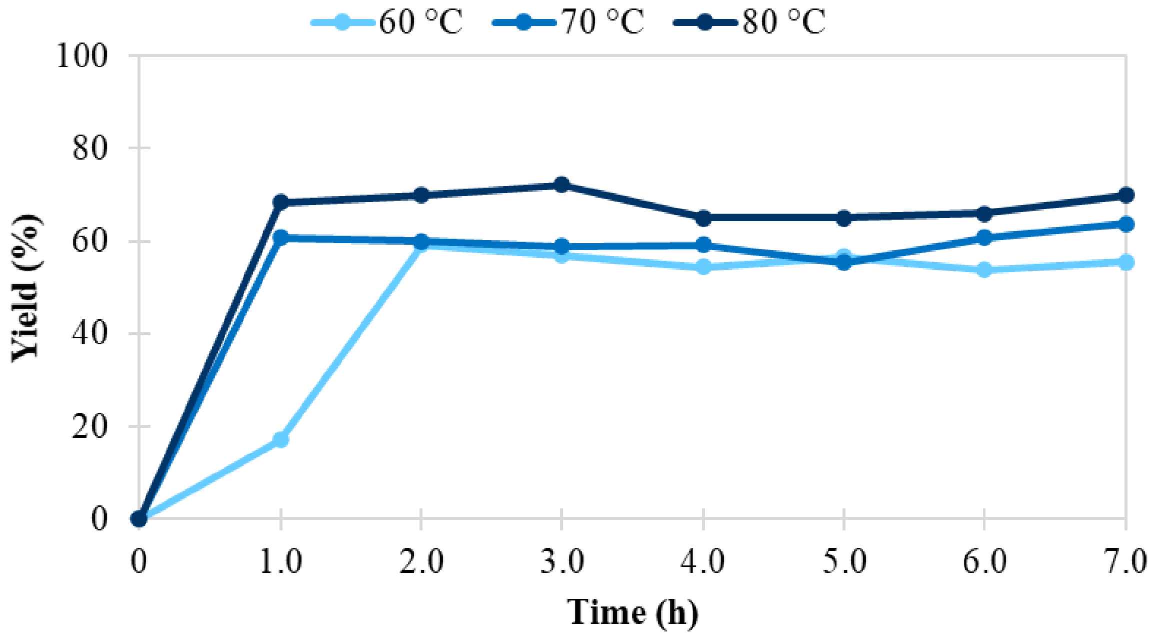

(**b**)

Figure S2. Ester yield (%) in the continuous enzymatic synthesis of *n*-amyl hexanoate in solventless system catalyzed by sol-gel entrapped *Candida antarctica* B lipase at: (**a**) a substrate ratio of 2:1 and a temperature of 60 °C; (**b**) a substrate ratio of 2:1 and a flow rate of 0.2 mL/min

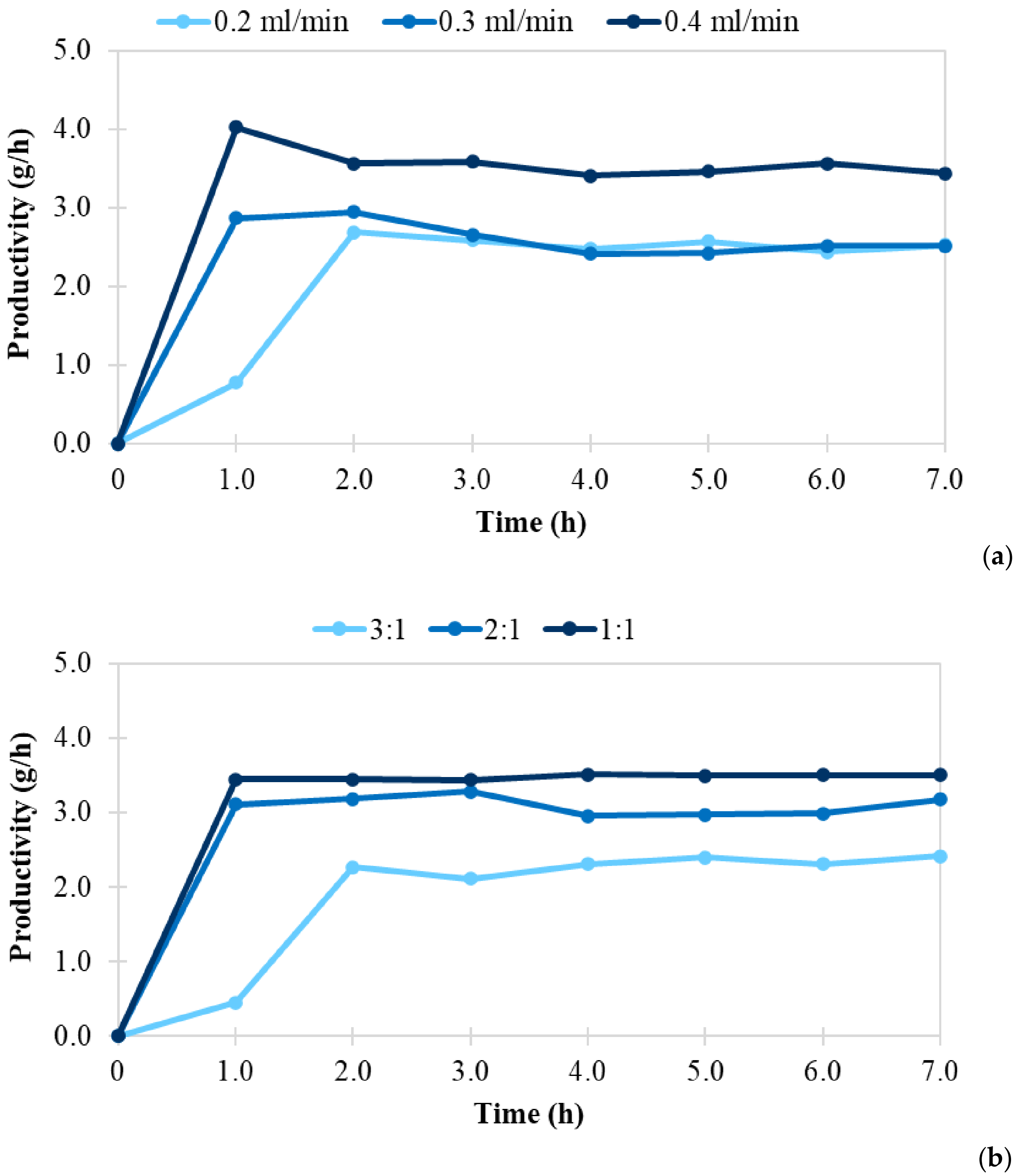

Figure S3. Productivity in the continuous enzymatic synthesis of *n*-amyl hexanoate in solventless system catalyzed by sol-gel entrapped *Candida antarctica* B lipase at: (**a**) a substrate ratio of 2:1 and a temperature of 60 °C; (**b**) a temperature of 80 °C and a flow rate of 0.2 mL/min
